# The mismatch negativity: a window to the brain

**DOI:** 10.3389/fnhum.2024.1499016

**Published:** 2024-11-20

**Authors:** Lilli S. Donovan

**Affiliations:** School of Medicine and Psychology, Australian National University, Canberra, ACT, Australia

**Keywords:** mismatch negativity, event-related potential, development, clinical, perception: auditory processing

In the late 1970s, the late Risto Näätänen and his colleagues conducted a series of event-related potential (ERP) studies to explore whether auditory-evoked potentials could provide evidence of “pre-attentive”—or preconscious—processing of unattended sounds. Their research led to the identification of a negative shift in the evoked potential waveform elicited by an infrequent, deviant stimulus when randomly and unpredictably presented among a sequence of repetitive, standard stimuli. This effect was later termed the “mismatch negativity” (MMN). These findings heralded a new era in cognitive neuroscience research, sparking vigorous scientific debate on the role of pre-attentive vs. attention-dependent processing, with MMN at the core of this discourse.

Research on the MMN has remained pertinent for almost 50 years. At the MMN2018 Conference, held at the University of Helsinki to celebrate the 40^th^ anniversary of MMN's discovery, it was highlighted that approximately 5,000 scientific reports have published mismatch negativity as a key topic, representing studies conducted in nearly 70 countries and in at least 11 different languages. Collectively, these studies have been cited over 145,000 times in more than 44,000 articles, reflecting MMN's relevance in fields as diverse as neuroscience, psychology, psychiatry, electrical engineering, artificial intelligence, and veterinary science.

The substantial volume of MMN research demonstrates its importance to both basic science and clinical settings. To synthesize the trends in MMN research into an accessible format and to provide newcomers with an up-to-date compendium of the field, Risto Näätänen, Teija Kujala, and Gregory Light recently published *The Mismatch Negativity: A Window to the Brain* (2019).

The book is structured into eight chapters, thoughtfully arranged to guide the reader from MMN's origins as a measure of auditory discrimination to its diverse applications across various psychological contexts. The chapters of the book can be broadly grouped into three thematic areas, making the content easy to navigate.

The first two chapters lay the groundwork for understanding MMN, tracing its origins as the first objective measure of auditory discrimination. These chapters discuss auditory change-detection research, providing detailed descriptions of the experimental paradigms designed to elicit the MMN response, the technical equipment necessary to record and observe it, and the brain regions responsible for its generation. The authors also offer a critical evaluation of the various—and at times conflicting—theories surrounding MMN generation, such as memory-trace formation, the degrees of refractoriness of N1 neurons, or a combination of both. The opening chapter extends this discussion to the biological systems that underpin sensory processing, including a notable section on MMN findings from animal studies, which have utilized alternative techniques like epidural recordings. In addition, the chapter covers the diverse array of brain imaging technologies employed in MMN research, from magnetoencephalography (MEG) and functional magnetic resonance imaging (fMRI) to positron emission tomography (PET) and optical imaging (OI). This breadth underscores the adaptability of MMN as a tool, capable of being indexed across multiple research methodologies.

While these introductory chapters provide rich insights, they presuppose a foundational understanding of ERP research. There is little discussion of the fundamentals of ERP techniques or explanations of how to interpret typical ERP data, which may present challenges for those entirely new to the field. However, for those with a working knowledge of ERP methodology, the chapters lay an exceptional groundwork for delving into the specifics of MMN, offering a solid starting point for further exploration into this field.

Chapters 3, 4, and 5 focus on MMN across the lifespan, examining its development from *in utero* through adolescence and into older adulthood. Chapter 3 begins with an overview of the developmental trajectory of MMN, with much of the research focusing on observations in infancy and changes to latency and amplitude of the MMN throughout childhood. The chapter also explores the remarkable perceptual abilities of infants, including their capacity to perform complex auditory tasks such as organizing sound streams, binding sound features, processing invariant speech characteristics, and extracting auditory rules from the environment. Chapter 4 continues to examine the variability in MMN amplitudes and latencies across development in the context of abnormal cognition, including dyslexia, specific language impairment, autism spectrum disorder, and oral clefts. The discussion points within this chapter center on diminished or enhanced amplitudes and delayed or accelerated latencies, providing insights into how these neurodevelopmental conditions influence MMN. Chapter 5 shifts focus to the effects of aging on MMN across auditory, visual, and somatosensory modalities. Notable topics include the age-related decline in auditory temporal resolution, the absence of MMN in response to demanding sequential stimuli, and the reduction in visual MMN amplitude when processing peripheral changes in the visual field.

These middle chapters prove especially valuable for seasoned researchers and clinicians eager to expand their grasp of MMN beyond its fundamental properties. They offer a deep dive into the developmental trajectory of MMN and its variability across diverse clinical populations, making them essential reading for those looking to enrich their expertise and explore the broader applications of MMN in both developmental and clinical contexts.

The final three chapters explore MMN deficits in a range of neuropsychiatric and neurological disorders, with a specific focus on its clinical application in predicting outcomes in altered states of consciousness. Notably, Chapter 6 details studies showing that the MMN is the earliest available indicator of awakening from coma, as well as a predictor of recovery of consciousness and a safeguard against the progression to a persistent vegetative state (PVS). Among the most compelling topics in this chapter is the changes that occur in the amplitude of the MMN prior to the recovery of consciousness and its implications for tracking and predicting recovery from PVS. The technical details of these studies are thoroughly explained, making it a valuable resource for researchers seeking to deepen their understanding of the complexities of coma-related MMN research. The chapter concludes with the discussion of an MMN-based bedside diagnostic tool developed to quantify consciousness levels at the bedside—a thoughtful addition for clinicians.

These chapters highlight the pivotal role of MMN in clinical research, illustrating its value as a robust indicator of cognitive and functional status across a wide range of disorders, regardless of their specific etiologies or symptomatic differences. The authors provide a nuanced exploration of the rationale behind each project, offering compelling insights into potential future avenues for MMN research. However, while these chapters present encouraging developments for the clinical application of MMN, they fall short in addressing some of the key limitations currently hindering its diagnostic use—such as the absence of standardized detection protocols and the need for more comprehensive formative data. Although a more detailed examination of these challenges would have been beneficial, the chapters still succeed in summarizing the substantial body of MMN research that has significantly advanced our understanding of neurological and psychiatric disorders. Ultimately, these final sections build a persuasive case that researchers and clinicians alike are on the cusp of translating MMN research into practical, clinical use.

The authors deliver a comprehensive exploration of both the fundamental and clinical applications of MMN, underscoring its relevance across the past several decades. The broad scope of the text ensures its appeal to a wide audience—from early-career researchers eager to grasp the basics to seasoned experts delving deeper into MMN's complexities. Crafted as an essential resource for ERP researchers, the book also invites scholars from a variety of disciplines to enrich their understanding of MMN's far-reaching implications.

MMN's potential as a diagnostic tool in clinical settings is made abundantly clear throughout the text. Several notable applications are highlighted, including the objective monitoring of sensory memory development in infants and children, tracking the age-related decline of these processes in older populations, predicting cognitive recovery following stroke or traumatic brain injury, and reading difficulties (e.g., dyslexia) through electrophysiological recordings in conjunction with objective assessments. The book also discusses MMN's role in evaluating cognitive and functional status in individuals with neurological disorders, such as schizophrenia, epilepsy, Alzheimer's disease, and Parkinson's disease, further demonstrating its invaluable utility in clinical practice.

Since the book's release, the MMN community has faced the profound loss of its pioneering figure, Risto Näätänen. His passing signifies the close of a remarkable chapter, but his colleagues and the rising generation of MMN researchers are poised to carry forward his legacy. Looking to the future, a subsequent edition of this book would benefit from reflecting on how MMN research has evolved since its publication and from offering further discourse on the future trajectory of the field. In the book's concluding remarks, the authors acknowledge that despite being heralded as a “breakthrough biomarker,” MMN has yet to gain widespread clinical adoption. This acknowledgment raises critical questions for the future of MMN research: What should the foremost objective of future investigations be? Should the focus shift toward closing the gap between laboratory findings and clinical applications? And if so, what collaborative efforts between researchers and clinicians are necessary to facilitate this transition?

In summary, *The Mismatch Negativity: A Window to the Brain* offers a superb entry point into MMN research. Näätänen's passion permeates every chapter, and the book delivers a meticulously structured overview of pivotal studies and the latest advancements in the field. It stands as an invaluable resource for scholars seeking to deepen their understanding of MMN's mechanisms and potential applications.

